# Inhibition of GSK3α/β impairs the progression of HNSCC

**DOI:** 10.18632/oncotarget.25250

**Published:** 2018-06-12

**Authors:** Lisa Schulz, Ralph Pries, Aruna Sree Lanka, Maren Drenckhan, Dirk Rades, Barbara Wollenberg

**Affiliations:** ^1^ Department of Otorhinolaryngology, University of Luebeck, Luebeck 23538, Germany; ^2^ Department of Radiation Oncology, University of Luebeck, Luebeck 23538, Germany

**Keywords:** HNSCC, GSK3

## Abstract

**Background:**

Head and neck squamous cell cancer (HNSCC) is one of the most common tumors worldwide and there is an enormous need for innovative therapy approaches. Several recent studies suggest tumor entity specific roles of glycogen synthase kinase 3 (GSK3) in different human cancers, acting as tumor suppressor or as tumor promoter. Here we describe the role of GSK3 with respect to different parameters within HNSCC progression.

**Methods:**

Base line expression and activity profiles of p-GSK3α/β (Ser21/9) and p-GSK3α/β (Tyr279/216) were analyzed by immunohistochemistry and western blotting. Four different permanent HNSCC cell lines were exposed to the potent GSK3α/β inhibitor SB 216763. Cell viability was controlled via the MTT test. Cell migration was quantified with the Real Time Cell Analyzer (RCTA) xCELLigence. Regulation of the epithelial-mesenchymal transition (EMT) was measured with the Human Epithelial to Mesenchymal Transition (EMT) RT^2^ Profiler™ PCR Array and scratch assays. Taqman probes were used to detect the specific gene expression profiles of inflammatory cytokines Interleukin IL1β, IL6, IL8, IL10, TNFα and IFNβ.

**Results:**

Exposure of permanent HNSCC cell lines to the specific GSK3α/β inhibitor SB 216763 leads to significant growth inhibition, inhibition of migration and decreased levels of active GSK3α/β in a dose dependent manner.

Exposure of HNSCC lines to SB 216763 also resulted in a markable shift of EMT markers and functional EMT dysregulation. Functionally GSK3 differentially mediates the expression of TLR4- and TLR3-induced inflammatory cytokines in HNSCC, whereas no effect of SB 216763 on the NFkB activity was noticed.

**Conclusion:**

GSK3α/β plays a crucial role in a variety of regulatory networks for HNSCC cancer progression as it drives proliferation or migration and thus GSK3 could serve as an interesting target for clinical drug development.

## INTRODUCTION

Head and neck squamous cell carcinoma (HNSCC) is one of the most common solid neoplasms worldwide. Its occurrence is being associated with exposure to smoking and alcohol consumption [1]. The mortality rates are still high due to local tumor invasion, development of metastases and failure of chemo- and radiation therapies [2–6].

Several recent studies suggest an active role of glycogen synthase kinase 3 (GSK3) in different human cancers, either as a tumor suppressor or as a tumor promoter [7].

GSK3 has been shown to play a central role in mediating anti-tumor immune responses, although the prominent role of GSK3 in the (APC)-beta-catenin destruction complex implies that an inhibition of GSK3 could trigger tumor promotion by activating β-catenin [8–9]. GSK3 is a ubiquitously expressed serine/threonine protein kinase, which can be found in all eukaryotes. There are two known isoforms of GSK3, GSK3a and GSK3β, which share 85% overall sequence homology, including 98% amino acid sequence identity within their kinase domains [8–12].

GSK3 has been found to phosphorylate almost 50 substrates from numerous different cellular pathways, which are involved in the regulation of various cellular processes such as differentiation, metabolism or apoptosis [13–19]. Unlike other kinases, the enzyme is constitutively active, merely being inactivated in response to cellular signals [20–22].

The best defined mechanism of GSK3 regulation is the phosphorylation of serine 21 (Ser21) in GSK3a or serine 9 (Ser9) in GSK3b, which leads to an inhibition of the enzyme activity. In contrast, the enzymatic activity is enhanced by phosphorylation of tyrosine279 (Tyr279) in GSK3a and tyrosine216 (Tyr216) in GSK3β, although the mechanisms regulating this modification are not fully understood yet [23]. The regulated phosphorylation of GSK3β (Ser9) is the main cause of various pathological conditions, and it has been proved to be upregulated in different epithelial cancers [17]. A further mechanism of the GSK3 regulation is the control of its intracellular localization and of its association in protein complexes, both serving to selectively direct the GSK3 actions towards specific substrates [18].

A strong inactivation of GSK3α/β has been suggested to support the malignant progression of different types of human cancer [7].

In this study, we first analyzed the tissue and cell distribution of cellular and subcellular GSK3α/β with its active and inactive isoforms. Secondly we clarified the functional meaning of the active GSK3α/β. Using the specific GSK3α/β inhibitor SB 216763 we comprehensively investigated the relevance of active GSK3 in proliferation, migration, EMT induction and finally TLR3- and TLR4-induced inflammatory responses of HNSCC.

## RESULTS

### GSK3α/β profiles in solid HNSCC and permanent cell lines

**Figure 1:**
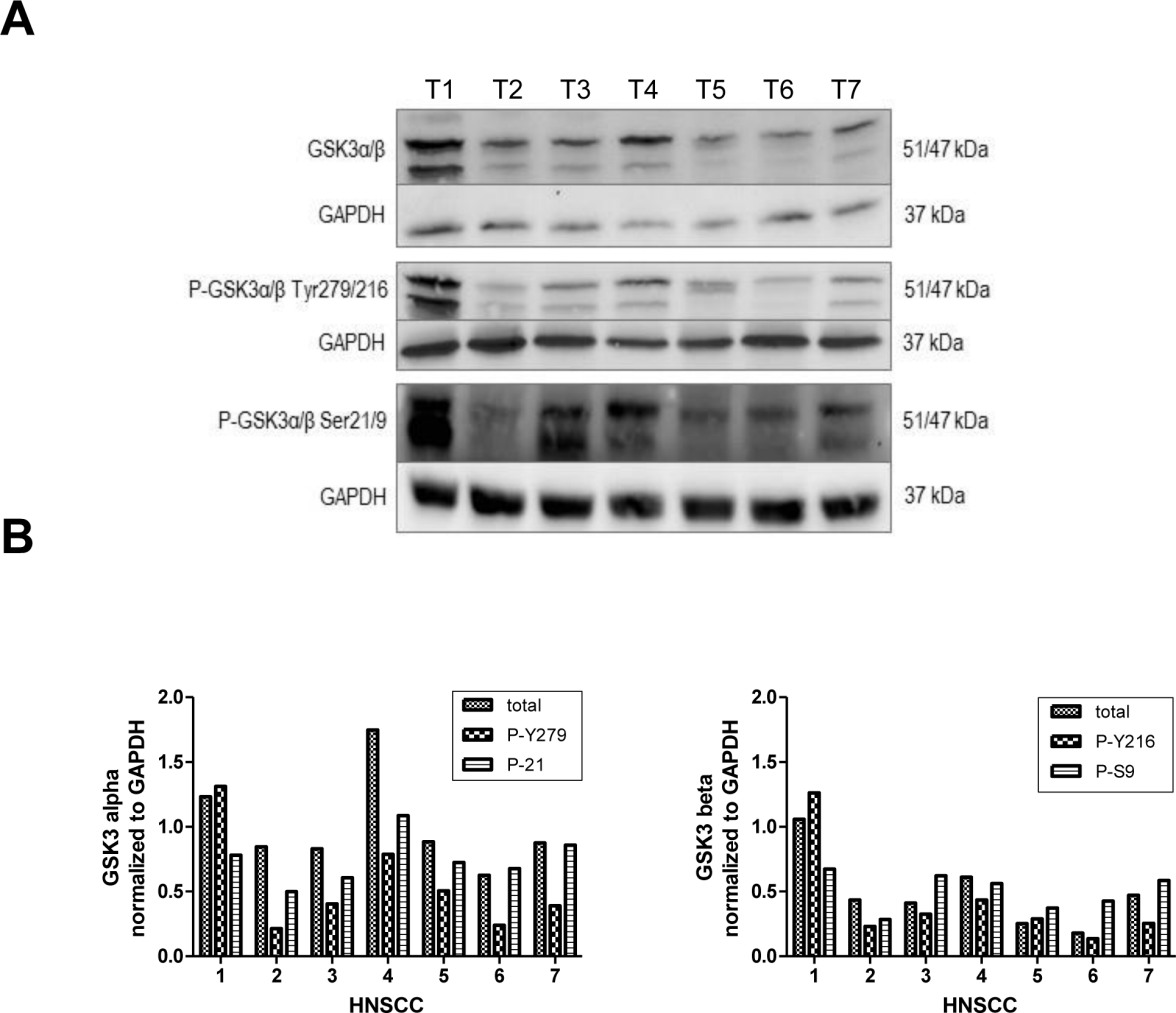
GSK3α/β expression profiles in solid HNSCC Western hybridization experiments were performed in order to analyze the expression and phosphorylation profiles of GSK3α/β in tissue samples of solid HNSCC. (**A**) Total protein levels of GSK3α/β were analyzed and in addition phosphorylation patterns of serine-21 in GSK3a or serine-9 in GSK3b as well as of tyrosine-279 in GSK3a and tyrosine-216 in GSK3β were investigated. (**B**) Quantification of GSK3α/β (Ser21/9) and GSK3α/β (Tyr 216/279) demonstrates a strong inhibition of GSK3α/β in all analyzed samples of HNSCC.

Western hybridization experiments were performed in order to analyze the expression and phosphorylation profiles of GSK3α/β in tissue samples of solid HNSCC. Our data demonstrate a heterogeneous expression of both GSK3 isoforms with respective phosphorylation patterns in the analyzed HNSCC tissue samples (Figure 1). The balance of active and inactive GSK3 represents a dynamic process and is determined by the individual characteristics and culture conditions of each cell line and each individual tumor. The expression levels of inactive GSK3α/β distinctly increased in comparison to the corresponding expression of its active counterpart in the same sample (Figure 1).

**Figure 2:**
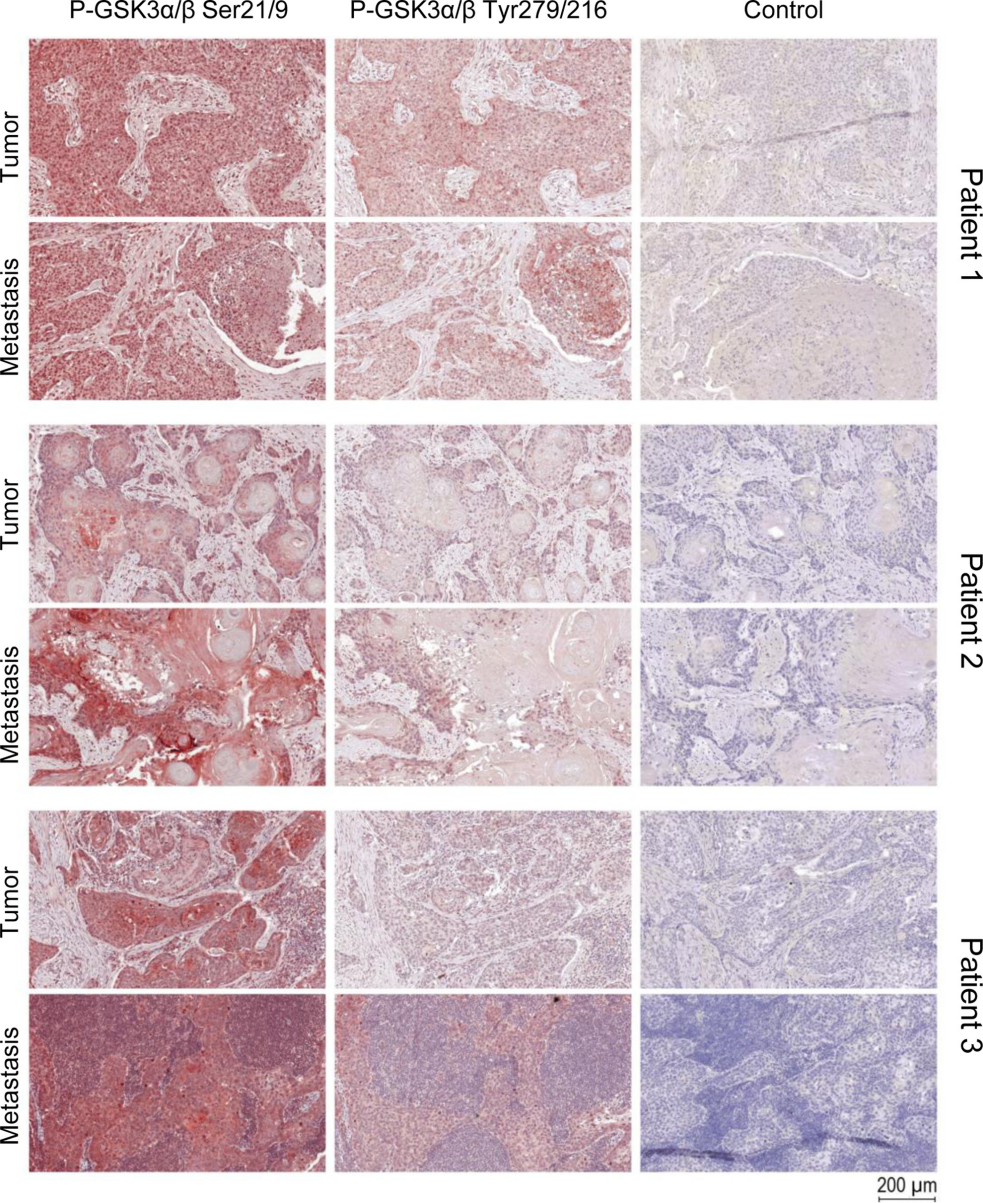
GSK3α/β staining in TMAs of HNSCC Immunostaining of tumor and metastases punches showed high expression of p-GSK3α/β (Ser21/9) and low expression of p-GSK3α/β (Tyr279/216), but both phosphorylated forms are expressed simultaneously. Cytoplasm and especially the nucleus showed a high expression of pGSK3α/β (Ser21/9). There is no difference between tumor and metastasis tissue regarding the expression of pGSK3α/β (Ser21/9) and pGSK3α/β (Tyr279/216).

**Figure 3:**
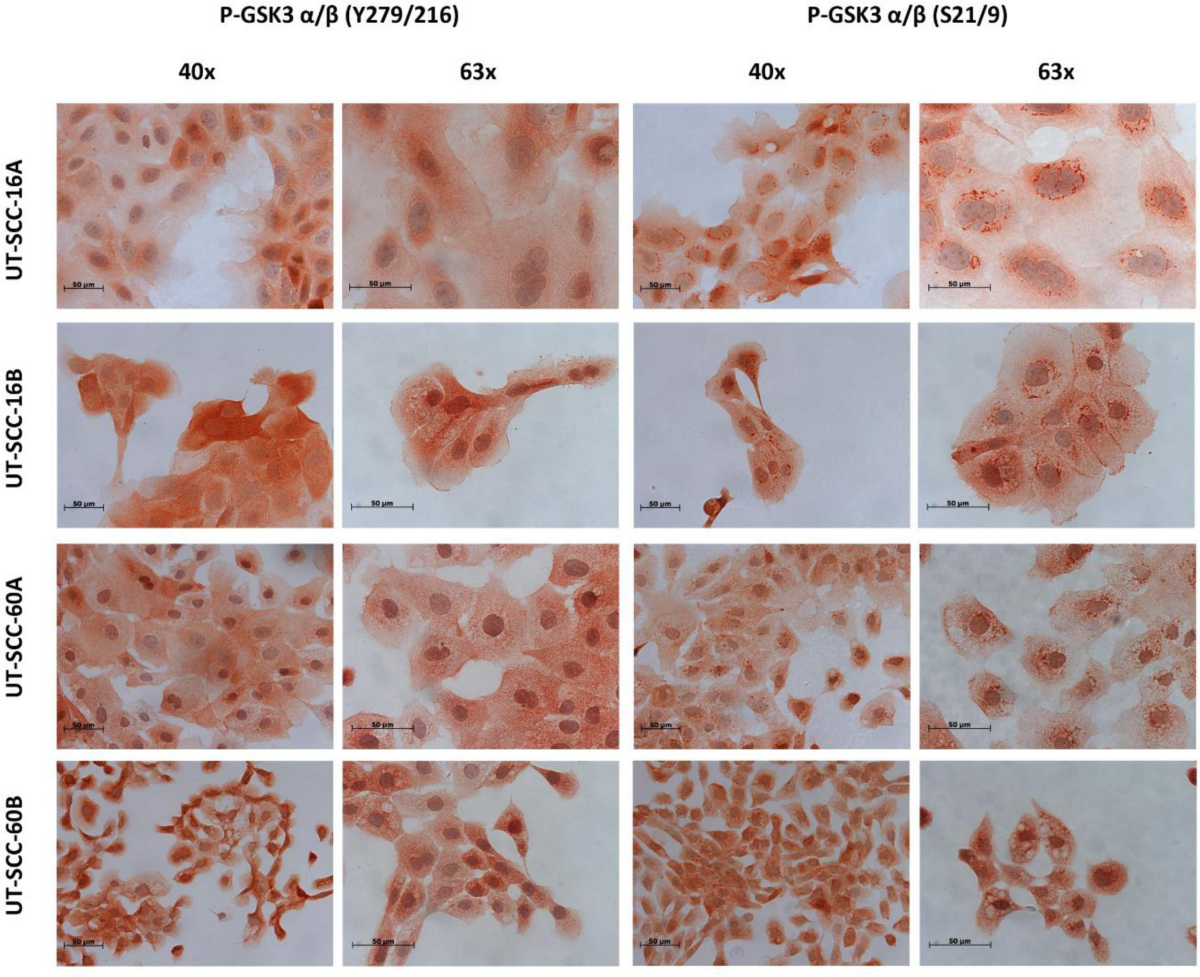
Immunohistochemical staining of p-GSK3α/β (Ser21/9) and p-GSK3α/β (Ser21/9) in permanent HNSCC cell lines LSAB staining of different permanent HNSCC cell lines showed a simultaneous expression of both phosyphorylated GSK3 forms. P-GSK3α/β (Tyr279/216) was to be found especially in the cytoplasm (homogenous distributed) and the nucleus. Staining of p-GSK3α/β (Ser21/9) showed a different subcellular localization. We could show an enrichment of p-GSK3α/β (Ser21/9) granularly around the nucleus in all examined HNSCC cell lines.

These findings were further corroborated by using immunohistochemistry experiments. Staining of active and inactive GSK3α/β in samples of solid HNSCC tumors and of the corresponding metastases as well as healthy tissue controls indicated a strong expression of inactive GSK3α/β (Figure 2). The subcellular localization of GSK3α/β in permanent HNSCC cell lines illustrated a homogeneous cytoplasmic localization of the active form in all analyzed samples, whereas inactive GSK3α/β was predominantly located in vesicle-like structures within the cellular lumen in addition to a cytoplasmic localization (Figure 3).

### GSK3 inhibition leads to a decreased migration activity and viability

**Figure 4:**
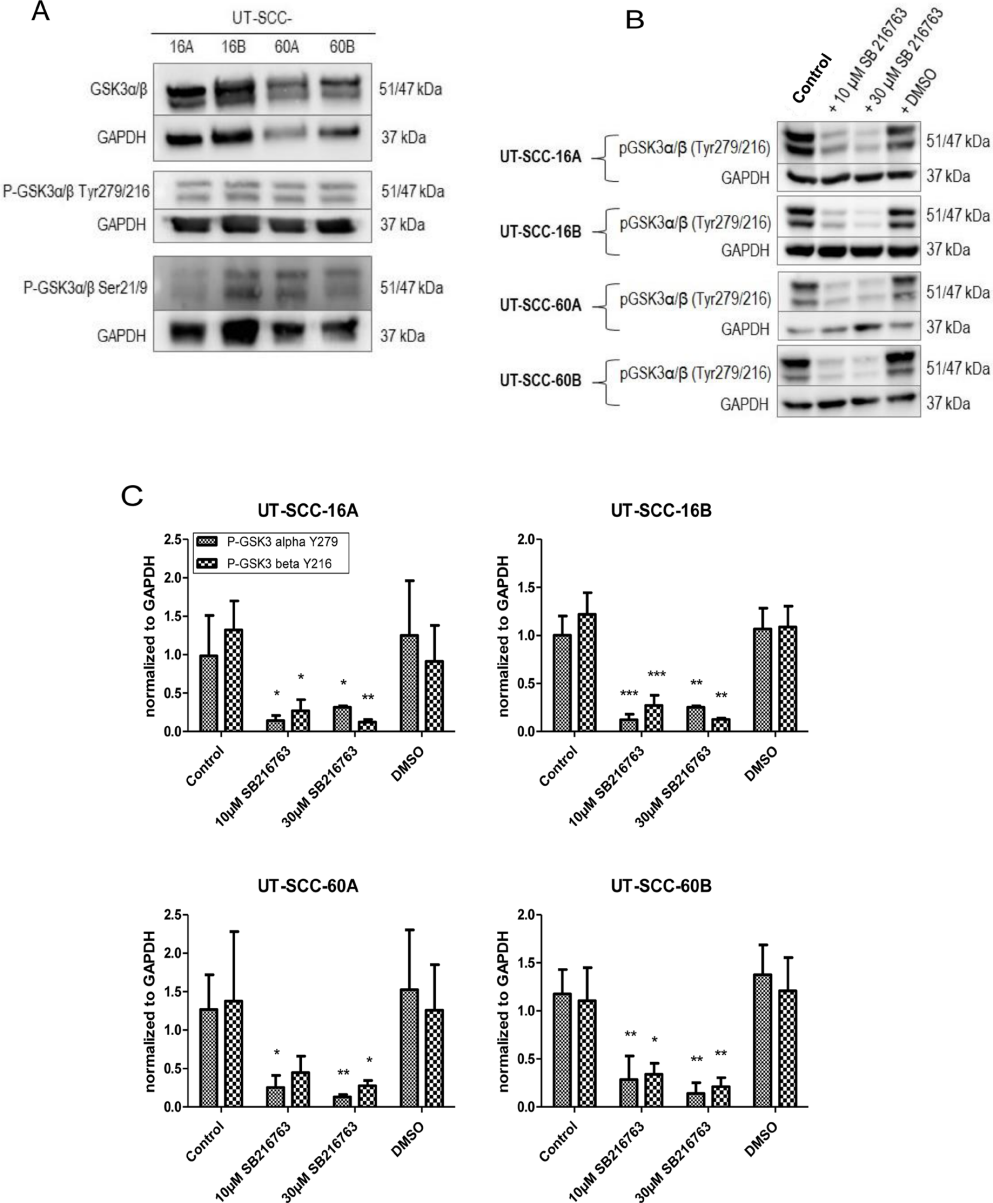
Decreased GSK3 activity in response to SB 216763 (**A**) Expression levels of total GSK3α/β, p-GSK3α/β (Tyr279/216) and p-GSK3α/β (Ser21/9) in permanent HNSCC cell lines UT-SCC-16A, UT-SCC-16B, UT-SCC-60 and UT-SCC-60B. (**B**) Cells of permanent HNSCC cell lines were incubated with 10 and 30 µM of the specific GSK3α/β inhibitor SB 216763, respectively. Our experiments revealed strongly decreased levels of active GSK3α/β in response to 24 hours of treatment with SB 216763 in a dose-dependent manner (**C**).

Expression analyses of total GSK3α/β, p-GSK3α/β (Tyr279/216) and p-GSK3α/β (Ser21/9) in permanent HNSCC cell lines UT-SCC-16A/B and UT-SCC-60A/B showed no correlation between the different expression levels (Figure 4A).

Analyzing the role of the remaining GSK3 activity in head and neck squamous cell carcinoma, cells were incubated with 10 and 30 µM of the specific GSK3α/β inhibitor SB 216763, respectively. Our experiments revealed strongly decreased levels of active GSK3α/β in response to 24 hours of treatment with SB 216763 in a dose-dependent manner (Figure 4B and 4C).

**Figure 5:**
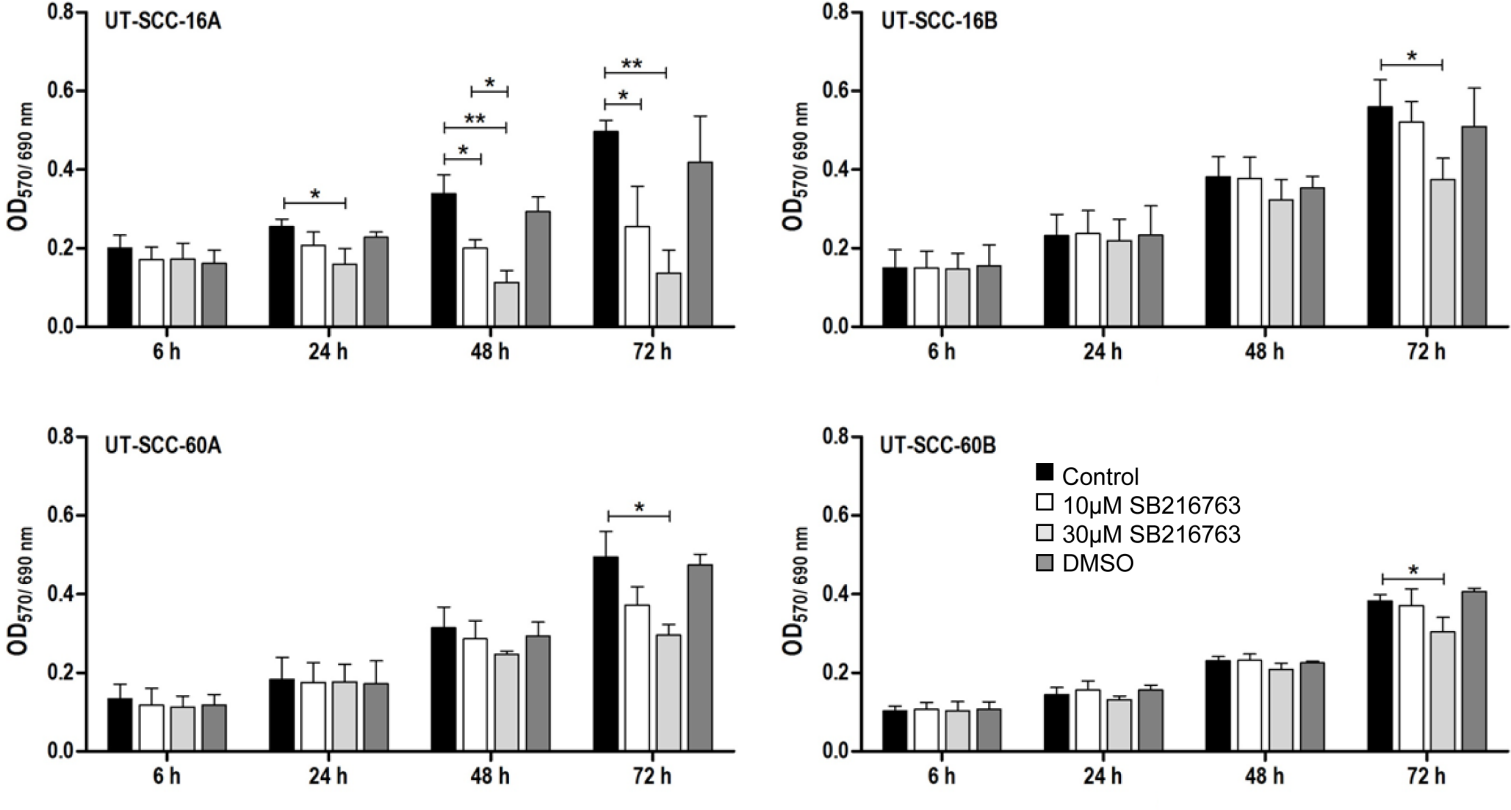
Influence of SB 216763 treatment on cell viability of permanent HNSCC cell lines measured by MTT Cytotoxicity Assay µM and 30 µM) by using MTT Cytotoxicity Assay. Bar graphs are showing changes in extinction over the different time periods. 24 hours after treatment, differences between the untreated control and treated cells showed up. Cell viability is significantly decreased after treatment of cells with the GSK3α/β inhibitor SB 216763 (30 µM) in all examined HNSCC cell lines after 72 hours. SB 216763 treatment had the most influence in UT-SCC-16A. DMSO had no effect on cell viability. Data are presented as mean ± SD (*n =* 3) of triplicate or quadruplicate experiments (^*^p < 0.05, ^**^p < 0.01).

MTT assays were carried out to measure the influence of the GSK3 inhibitor SB 216763 on the cellular viability. Our data reveal decreasing viabilities of the cell lines UT-SCC-16A/60A after 48 and 72 hours of respective incubation, whereas the cell lines UT-SCC-16B/60B appeared to be less sensitive in response to SB 216763 (Figure 5).

Furthermore, using the xCELLigence Real Time Cell Analyzer, we investigated the influence of the GSK3α/β inhibitor on the cellular migration activity of HNSCC cells.

**Figure 6:**
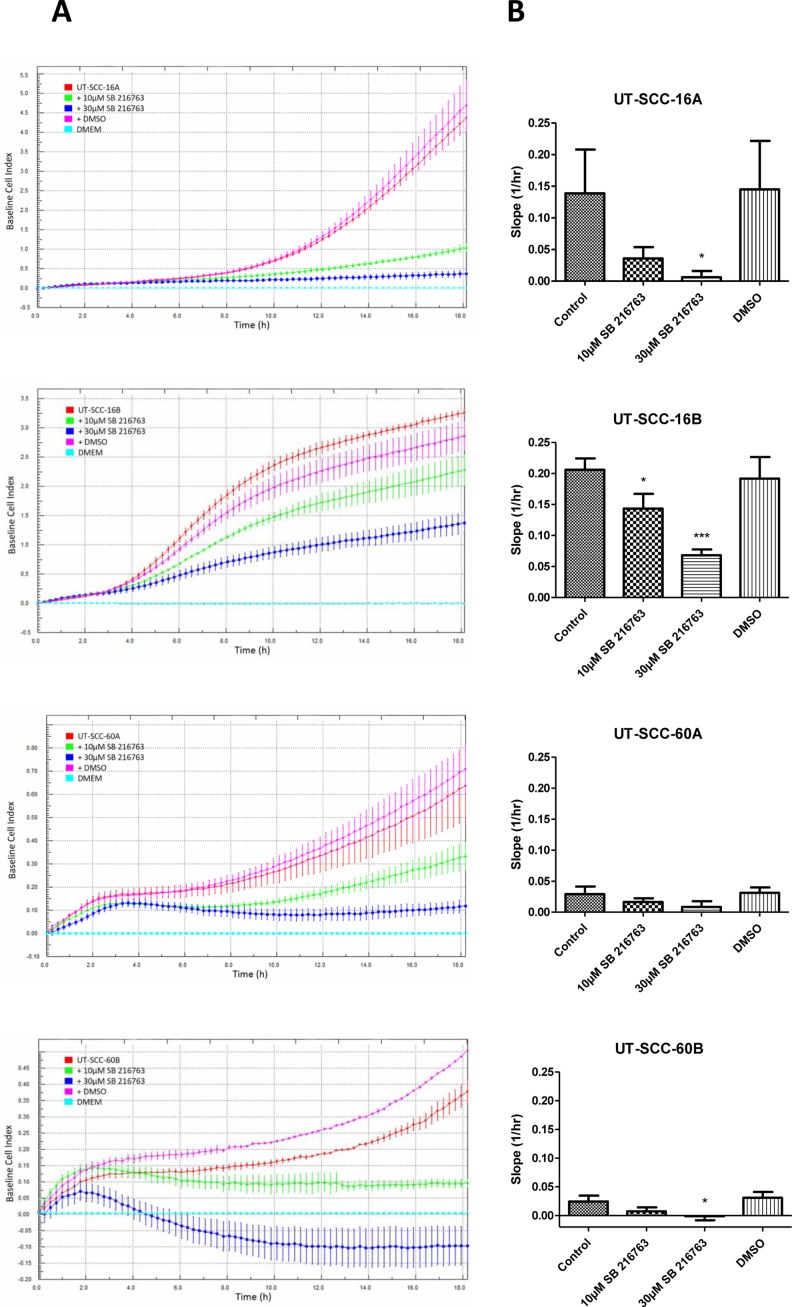
Real-time measurement of cell migration in permanent HNSCC cell lines Left column (**A**): Cell migration was analyzed for a time period of 18 hours, when cells were treated either with 10 µM or 30 µM SB 216763. For vehicle control, cells where treated with DMSO only. The treatment was done right before the measurement started. The results show, that cell migration is decreased, when cells are treated with the GSK3α/β inhibitor. We could show that for the cell lines UT-SCC-16A, UT-SCC-16B, UT-SCC-60 and UT-SCC-60B. The migration inhibiting effect was concentration dependent in all cell lines. DMSO had either no or only little effect on migration activity. Data are shown as mean ± SD of one representative experiment (three or four replicates). Right column (**B**): Bar graphs showing significantly decreased slope of Baseline Cell Index curve in UT-SCC-16A/B and UT-SCC-60B, when cells are treated with the GSK3 inhibitor (30µM). In UT-SCC-16B we could also show a significantly decreased slope after treatment with 10µM SB 216763. Data are presented as mean ± SD (*n =* 3) of triplicate or quadruplicate experiments (^*^p < 0.05, ^**^p < 0.01, ^***^p < 0.001).

**Figure 7:**
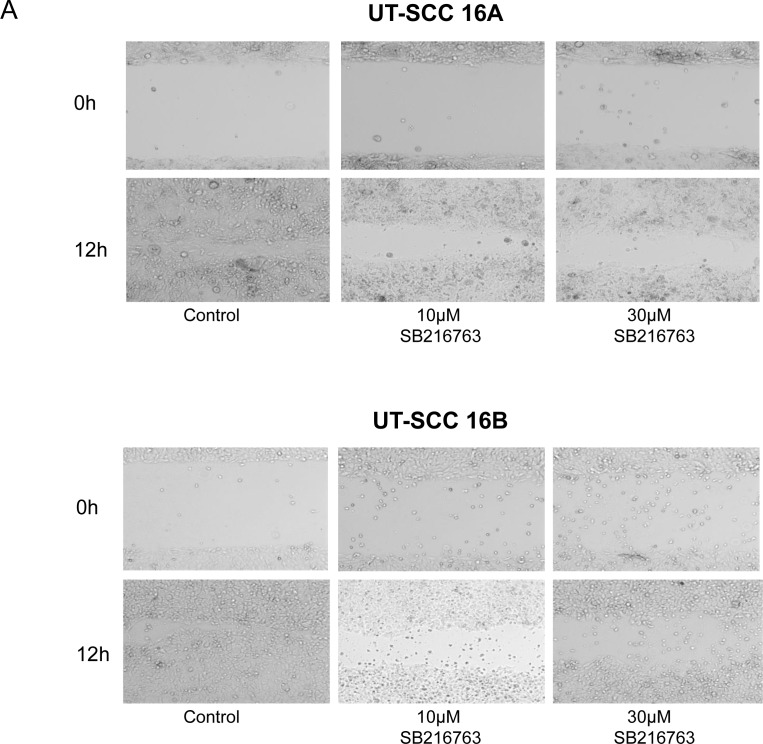

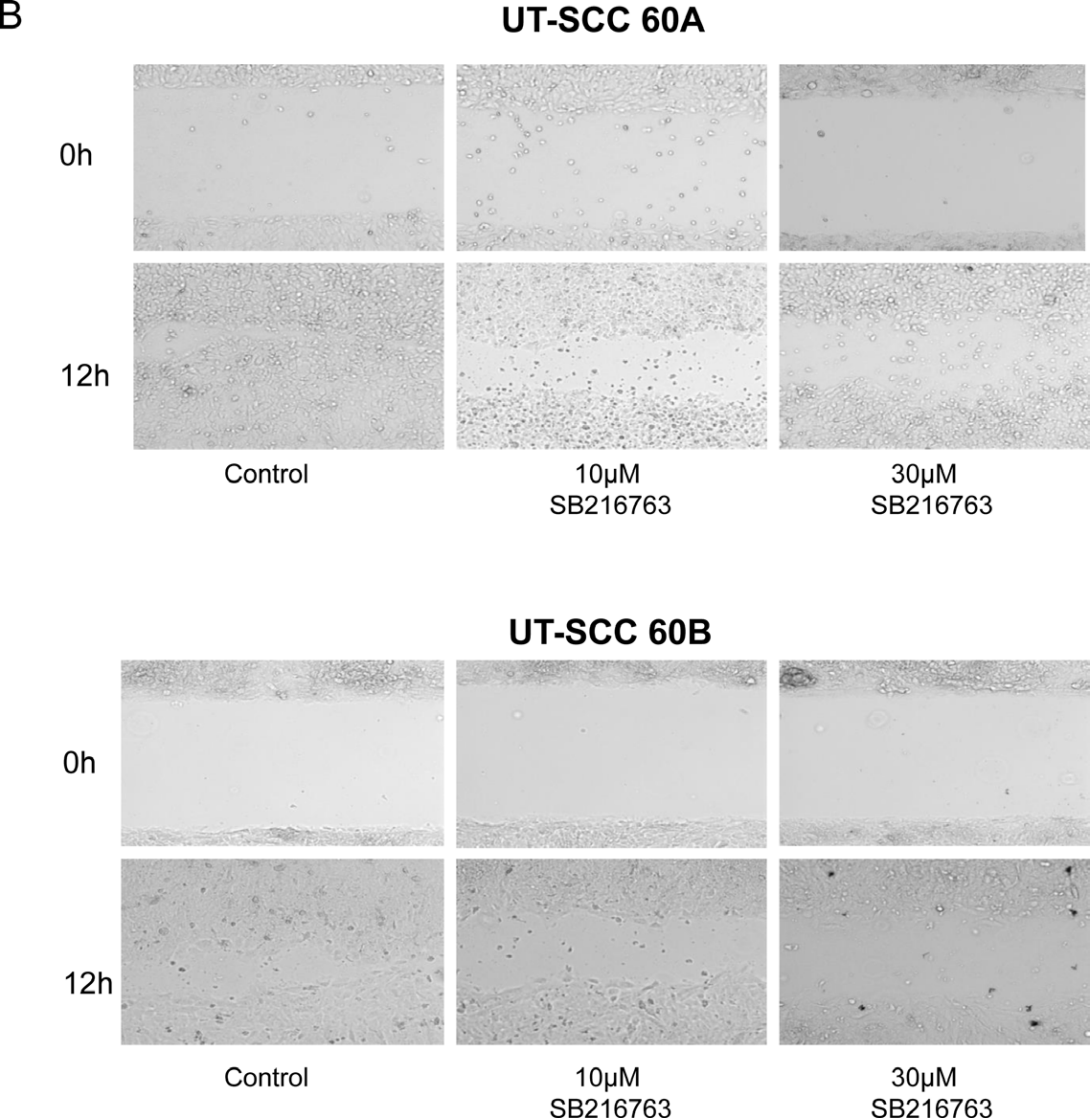
Scratch assay of permanent HNSCC cell lines after SB 216763 treatment Scratch assays of the HNSCC cell lines UT-SCC-16A and UT-SCC-16B (**A**) as well as UT-SCC-60A and UT-SCC-60B (**B**) showed a decreased migration activity in SB 216763 treated cells. The scratch was nearly closed after 12 hours in untreated cells. In treated cells there was still a gap left after the same time. The migration activity was concentration dependent. The higher the SB 216763 concentration, the bigger was the gap left after 12 hours. Treatment with DMSO had no effect on migration activity (data not shown). One representative experiment out of three independent experiments is shown.

A markedly decreased, dose-dependent cellular migration could be observed in response to an inhibition with SB 216763 for 24 hours (Figure 6). These results were underlined by *in vitro* scratch assay experiments, which indicate a strongly decreased cellular migration activity in response to SB 216763 (Figure 7).

### EMT dysregulation in response to SB 216763

The Wnt/ß-catenin signalling is well known to be involved in the regulation of the epithelial-mesenchymal transition (EMT). We investigated the transcriptional profiles of various EMT related genes in response to SB 216763 in order to asses the possibility of a redifferentiation with respect to an inhibition of the residual GSK3 activity. Therefore we used the PCR based ‘RT^2^ EMT Profiler’, which allows the comprehensive analyses of 84 different EMT related genes. The received data revealed strongly modulated mRNA levels of various EMT related genes in response to SB 216763 in the analysed HNSCC cell lines.

**Figure 8:**
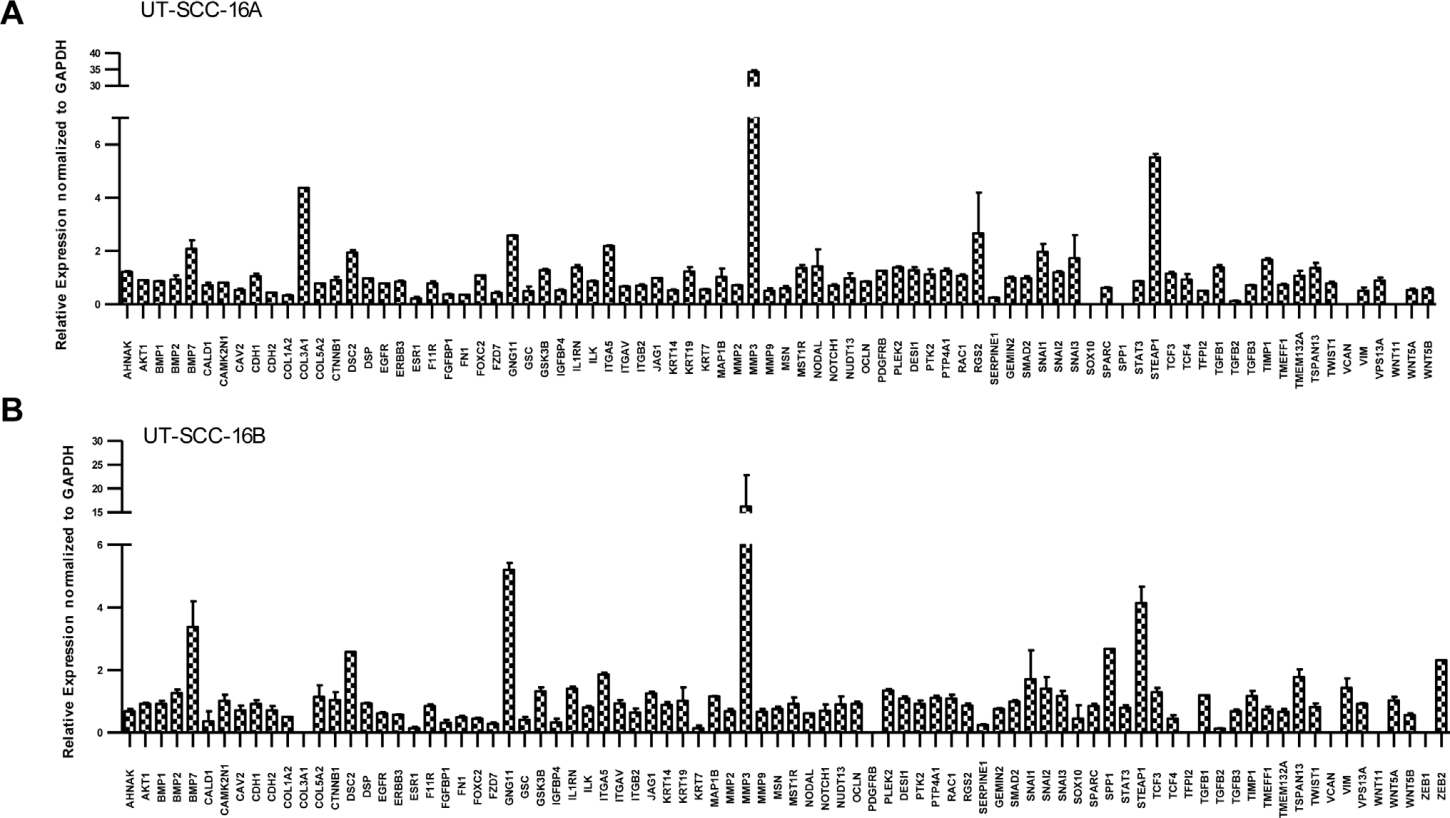
Dysregulation of EMT in response to SB 216763 Transcriptional profiles of various EMT related genes in response to the GSK3α/β inhibitor SB 216763. 84 different EMT related genes are shown in permanent HNSCC cell lines UT-SCC 16A/B (**A** and **B**) in response to SB 216763. Strongly increased mRNA levels of the EMT related genes BMP7, DSC2, GNG11, MMP3 as well as STEAP1 are illustrated. BMP7 as well as DSC2 are supposed to act as negative EMT regulators, whereas GNG11, MMP2 and STEAP1 are suggested to be activators.

The permanent HNSCC cell lines UT-SCC-16A/B showed strongly increased mRNA levels of the EMT related genes BMP7, DSC2, GNG11, MMP3 as well as STEAP1 (Figure 8). BMP7 as well as DSC2 are supposed to act as negative EMT regulators, whereas GNG11, MMP2 and STEAP1 are suggested to most likely activate EMT. Thus our data indicate a strongly dysregulated EMT regulatory network in response to a dysregulated GSK3 activity.

### GSK3 differentially modulates TLR-mediated cytokine expression

**Figure 9:**
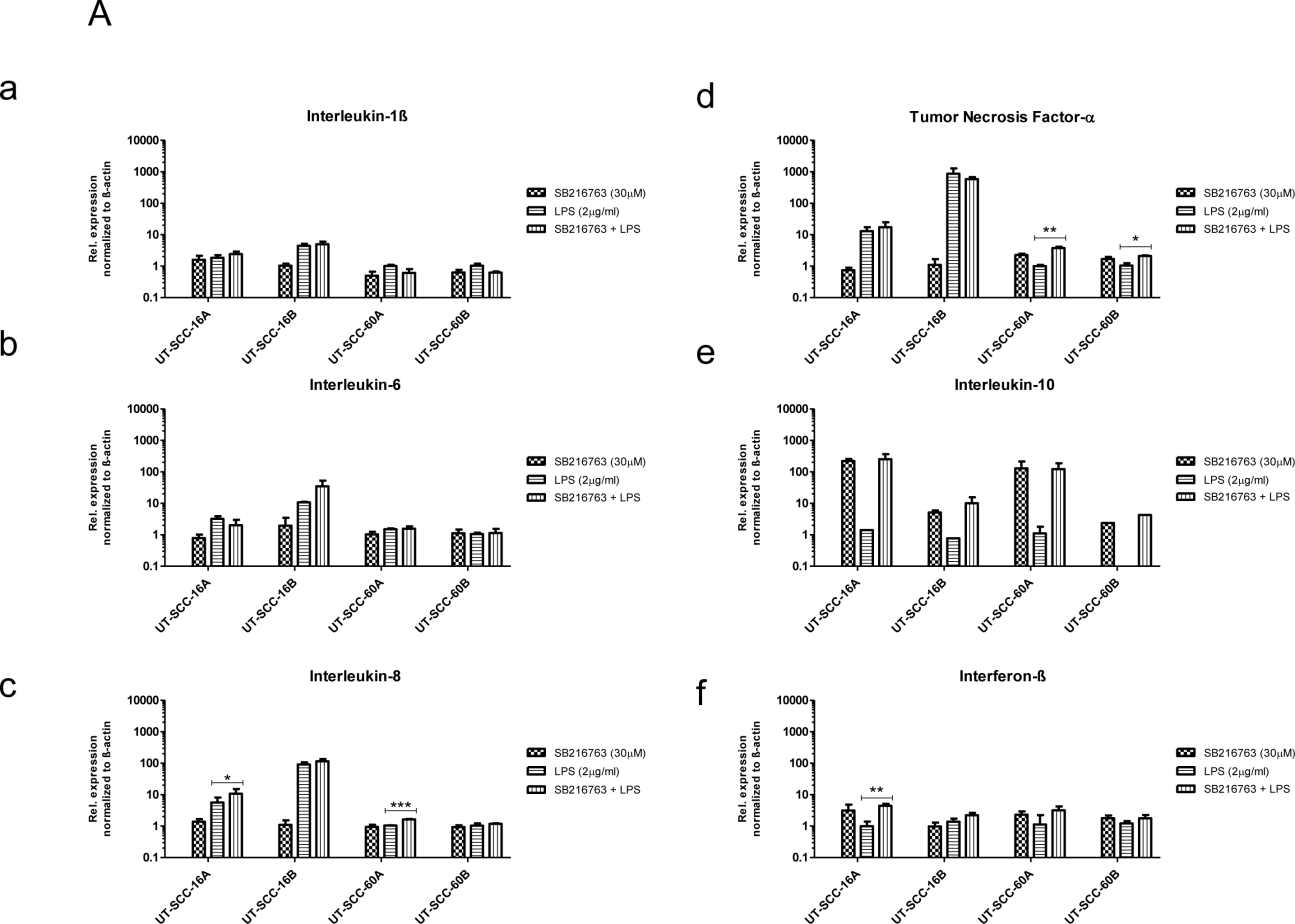

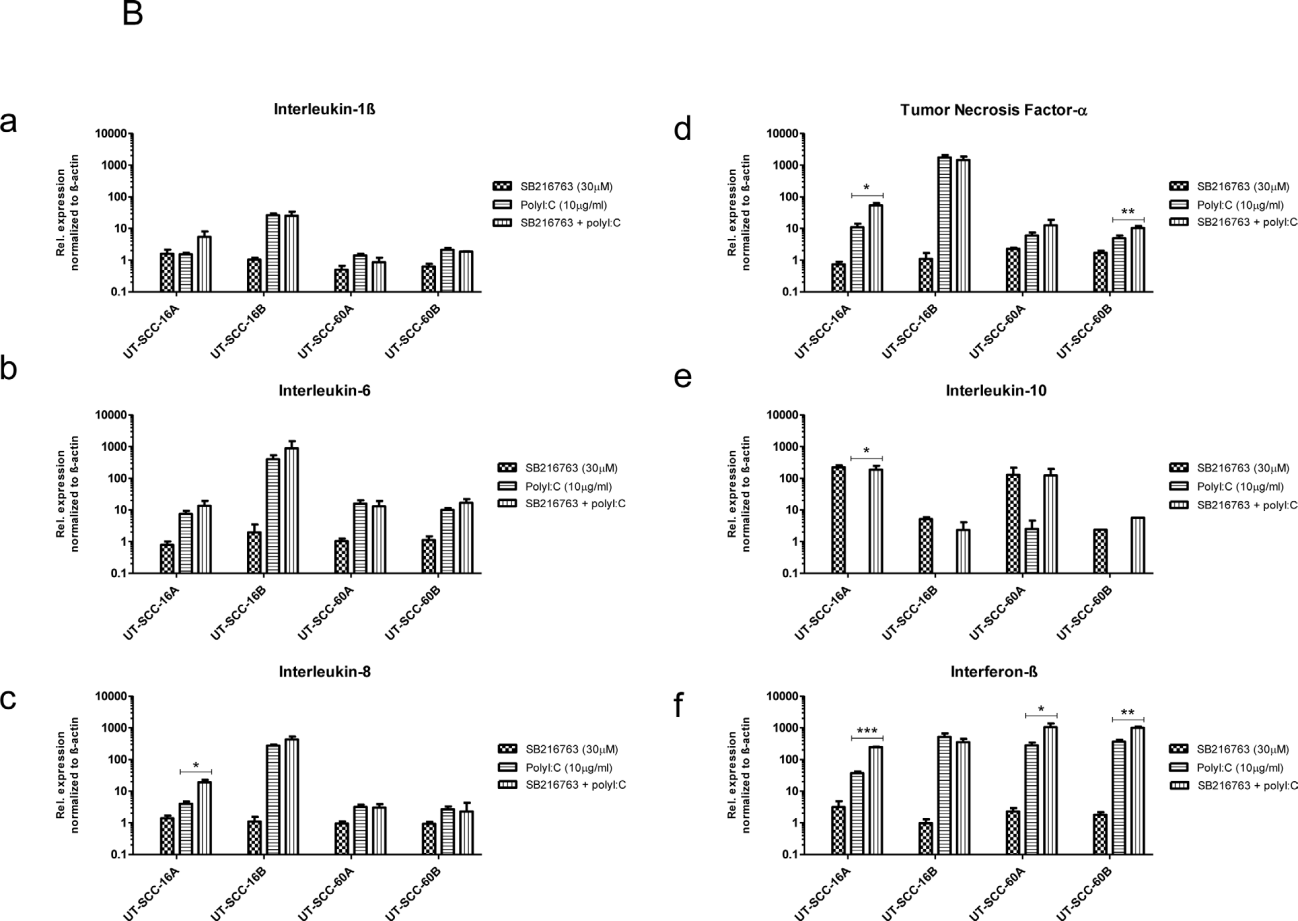
Influence of GSK3α/β inhibition on TLR-induced cytokine expression Differential modulation of TLR4- (**A**) and TLR3- (**B**) induced inflammatory cytokine expression in response to GSK3α/β inhibition. Strong induction of Interleukin (IL10) (e) expression in both LPS and Poly (I:C) stimulated cell cultures in the presence of GSK3 inhibitor. Slighter increased in the expression of IL8 (c), TNFα (d) and IFNβ (f) was noticed. Whereas, the expression of TLR4- and TLR3-mediated IL1β (a) and IL6 (b) was not clearly influenced by the inhibition of GSK3. Relative fold expression was normalized to β-actin. ^*^P ≤ 0.05; ^**^P ≤ 0.01; and ^***^P ≤ 0.001, compared with LPS stimulation (a–f) or Poly (I:C) stimulation (a–f). Results represent mean ± SD of three independent experiments.

Although GSK3 has been shown to be critical in regulating a variety of cellular functions, there is little known about the ability of GSK3 to regulate TLR-mediated inflammation in HNSCC. Thus, we investigated the role of GSK3 in regulating the expression of TLR3 and TLR4 mediated pro- and anti-inflammatory cytokines (Figure 9). The GSK3 α/β inhibitor SB 216763 alone had no apparent effect on the expression of pro-inflammatory cytokines IL1β, IL6, IL8, TNFα and IFNβ compared to untreated controls. Combining SB 216763 and TLR agonistic treatment with LPS or Poly (I:C) lead to markable changes in the cytokine production. Expression of the anti-inflammatory cytokine IL10 was increased by ten to five hundred fold and a slight increase in pro-inflammatory cytokines IL8, TNFα and IFNβ was found compared to cultures exposed to TLR agonistic treatment alone.

No significant effect was noticed in the expression of IL1β and IL6 (Figure 9).

To elucidate whether the activity of the transcriptional activator NFkB is affected in response to SB 216763, HEK-Blue™ TLR3 cells as well as HEK-Blue™ TLR4 cells were used. These cells were obtained by co-transfection of the human TLR3 or TLR4 gene and an inducible SEAP (secreted embryonic alkaline phosphatase) reporter gene into HEK293 cells. The SEAP gene is placed under the control of the IFNβ minimal promoter fused to five NF-κB and AP-1-binding sites. Stimulation with the TLR3 ligand Poly(I:C) or the TLR4 ligand LPS activates NF-κB and AP-1 which induce the production of SEAP, which can be easily determined using the HEK-Blue detection medium. Our data clearly indicate, that SB 216763 has no effect on the TLR3 or TLR4 triggered activation of NFkB (data not shown).

In summary, these data demonstrate that inhibition of the residual active GSK3α/β by SB 216763 differentially modulates the progression and cytokine expression profiles of HNSCC cells, independently of TLR3 and TLR4 induced NFkB signalling.

## DISCUSSION

GSK3 is an enzyme involved in regulating growth, cell cycle progression, apoptosis, and invasion. The deregulation of GSK3 is involved in several types of human cancer [23]. Hence, it has been suggested as an ideal therapeutic target against cancer progression [24] due to its role in both the extrinsic and the intrinsic apoptotic pathways and due to the fact that active GSK3 is nontoxic to non-cancerous cells [25]. There has been a number of conflicting reports concerning the extent of tumor progression in context with the expression of total GSK3β in human cancers [26, 27]. Our data revealed a strong inactivation of GSK3α/β in permanent HNSCC cell lines as well as in solid HNSCC, which has also been reported for other types of cancers. A strong inactivation of GSK3 has been suggested to support the malignant progression in oral cancer [11], whereas the potential relevance of active GSK3 has been mainly neglected.

Therefore, we used the specific GSK3α/β inhibitor SB 216763 to investigate the relevance of the remaining active fraction of GSK3α/β in the progression of HNSCC. colorectal cancer [27], and myeloma [28], depicting partially positive outcomes. Inhibition of GSK3 has been shown to be a double-edged sword with both tumor suppressing and tumor promoting properties [7].

Our data show a well bipolar impact of SB 216763 in HNSCC cell cultures. Inhibition of GSK3 leads to reduced cell migration activities and cellular viability rates indicating the crucial function of GSK3 in HNSCC progression. We also found that inhibition of GSK3 function strongly induced the expression levels of immunosuppressive cytokine IL10 and the activity of β-catenin.

It has been suggested that the observed discrepancy concerning the role of GSK3 as a tumor suppressor and tumor promoter depends on the specific cell type as well as on the signalling microenvironment. In prostate cancer, GSK3 has been shown to inhibit androgen receptor-stimulated cell growth, whereas high expression levels of GSK3 have been shown to participate in NF-*κ*B mediated cell survival in pancreatic cancer [29, 30]. Indeed, several studies also suggest that GSK3 exerts dual effects on NF-κB activity either suppressing or enhancing the inflammatory responses [33]. A well-known fact of phospho-inactivation of GSK3 is its ability to suppress MyD-88 cytokine production. The consequence is suppression of MyD-88 dependent cytokine production and enhancement of MyD88 independent cytokine production [34]. A recent study reported that inhibition of GSK3 leads to increased production of TLR4 induced IFNβ in macrophages [34]. Our studies demonstrate identical effects of GSK3 in mediating the expression of TLR4- and TLR3- induced inflammatory cytokines. Inhibition of GSK3 augments the strongly increased expression of anti-inflammatory cytokine IL10 as it has been described before in naive murine CD4 (+) T cells, where GSK3 inhibitors dramatically increased production of IL-10 [35]. The expression levels of pro-inflammatory cytokines IL8, TNFα and IFNβ were as well increased, whereas the expression of other pro-inflammatory cytokines such as IL1β and IL6 was not strongly affected. These data demonstrate that GSK3 inhibition can modulate differentially the nature of both pro- and anti-inflammatory mediators leading to either promotion or suppression of HNSCC cell growth. Besides its role in TLR mediated signalling cascades, GSK3 is also known to be involved in the regulation of retinoic acid inducible gene 1 protein (RIG1)-like receptor signalling [36]. GSK3 is also known for its ability to either activate or inhibit apoptosis, which underlines the complex regulatory network of this kinase in human cancers [24].

It is well known that GSK3 has a huge variety of phosphorylation targets, so that the overall effect of GSK3 regulation on cancer progression is determined by various aspects such as cell type, transformation status, and the specific signalling pathways being activated. In neuroblastoma cells for example, GSK3 has a substantial pro-apoptotic function, but it is the inhibition of GSK3 which finally results in a massively decreased cellular viability [31]. In this case, it has been shown that the inhibition of GSK3 can serve to tip the balance in favour of apoptosis, like it has as well been demonstrated in murine xenograft models for neuroblastoma and glioma [31, 32, 37]. It has been shown in this work, that the EMT status is influenced by GSK3 in HNSCC. The EMT status of tumor cells is strongly associated with disease progression, chemoresistance and clinical outcome. This includes the expression of characteristic EMT marker proteins and transcription factors, the migratory and proliferative activity of cells as well as the activity of important cellular signalling pathways involved in EMT [38].

The bifunctional role of GSK3 as a facilitator of apoptosis and a mediator of pro-survival signals underlines the importance of a fine tuned balance of active and inactive GSK3 to ensure the progression of HNSCC and could be a suitable target for clinical intervention.

## MATERIALS AND METHODS

### Cells and cell culture conditions

The adherent human head and neck squamous cell carcinoma cell lines UT-SCC-16A, UT-SCC-16B, UT-SCC-60A and UT-SCC-60B (UT-SCC-16A/B: female patient, tongue SCC (A) and cervical lymph node metastasis (B), PMID 9187133, 15287027 - UT-SCC-60A/B: male patient, HNSCC (A) and cervical lymph node metastasis (B), PMID 15287027) were a generous gift from Reidar Grenmann (University of Turku, Finnland). Cells were maintained in Dulbecco’s modified Eagle’s medium (DMEM,4,5 g/l D-glucose and L-glutamine) supplemented with 10% fetal bovine serum (FBS) (both Life technologies, Carlsbad, CA, USA),1% Sodium Pyruvate (PAN-Biotech, Aidenbach, Germany) and 1% antibiotics (10.000U/ml penicillin, 10.000 µg/ml streptomycin) (Biochrom, Berlin, Germany) at 37° C under 5% CO_2_ and 95% air atmosphere.

The HEK293-derived HEK-Blue hTLR3 cells as well as HEK-Blue hTLR4 cells containing a secreted embryonic alkaline phosphatase (SEAP) reporter gene, used for measuring TLR3/TLR4 activation (InvivoGen, San Diego, CA, USA) were cultured in HEK-Blue Detection medium following the manufacturer’s protocol.

### Reagents

The specific GSK3α/β inhibitor SB 216763 (10 and 30 µM, Sigma-Aldrich St. Louis, MO, USA) is a structurally distinct maleimide which inhibits GSK3α *in vitro* in an ATP-competitive manner with an IC50 value of 34 nM. It also inhibits GSK3β with a similar potency. The selectivity for GSK3α/β was established by testing SB 216763 against a panel of 24 protein kinases, including PKB and 3-phosphoinositide dependent protein kinase-1 (PDK-1) Dimethylsulfoxid (DMSO) (Sigma-Aldrich, St. Louis, MO, USA) was used as vehicle control in all experiments.

### Antibodies

Anti-phospho-GSK3α/β (Ser21/9) (#9331) and anti-GAPDH (#2118) (Cell Signaling Technology, Danvers, MA, USA), anti-Phospho-GSK3α/β (Y279/Y216) (#ab68476, Abcam, Cambridge, UK) were used in a 1:1000 dilution in TBS containing 3% BSA (PAA Laboratories, Pasching, Austria). Anti-active-β-Catenin (#05-665) (Merck Millipore, Billerica, MA, USA) was used in a 1:2000 dilution in TBS and 3% skim milk (Carl Roth, Karlsruhe, Germany). The species specific secondary antibodies anti-mouse IgG (whole molecule)–peroxidase (# A9044) and anti-rabbit IgG (whole molecule)–peroxidase (#A0545) (Sigma-Aldrich, St. Louis, MO, USA) were used in a 1:50.000 dilution in TBS containing either 3% BSA or 3% skim milk.

### Western hybridization

Cells were washed twice in ice-cold PBS and then lysed in RIPA-Buffer (Cell Signaling Technology, Danvers, MA, USA) containing aprotinin, pepstatin A, Protease Inhibitor Cocktail and phenylmethanesulfonyl fluoride (Sigma-Aldrich, St. Louis, MO, USA) for 1.5 hours. Cellular debris were removed by centrifugation (13.000 rpm, 4° C, 10 min). Protein concentration was determined using Bradford assay with Quick Start™ Bradford 1× Dye Reagent (Bio-Rad Laboratories, Hercules/CA, USA). 4× SDS sample buffer was added and the samples were boiled at 95° C for 5 min. Equivalent amounts of protein (30 µg) were separated by SDS-PAGE in a 10% polyacrylamide gel and transferred onto nitrocellulose membranes by tank-blotting. For protein extraction from tissue, the tissue was homogenized with 500 µl RIPA buffer and protease inhibitors by using TissueLyser LT (Qiagen, Hilden, Germany) and then incubated and centrifugated as mentioned above.

The membranes were blocked with TBS containing 3% BSA or 3% skim milk for 1 hour at room temperature and then incubated with the primary antibodies overnight at 4° C. After washing with TBS, the membranes were incubated with the species specific secondary antibody for 1 hour at room temperature. The protein bands were visualized using Amersham™ ECL™ Primer Western Blotting Detection Reagent (GE Healthcare, Chalfont St Giles/Buckinghamshire, UK) and Fusion-FX7 as well as the corresponding software Fusion Bio-1D V12.14 (VilberLourmat, Eberhardzell, Germany), after extensive washing. The nitrocellulose membranes were re-probed overnight at 4° C with GAPDH antibody. Afterwards the membranes were washed, incubated with secondary antibody and ECL detection reagent again.

### Immunohistochemistry

Tissue slides were deparaffinized with xylene twice for 10 min each, and rehydrated with alcohol in four baths of decreasing concentrations (100%, 96%, 70% and 50%) for 3 min. each, followed by washing with distilled water and rinsed in TBS three times for 3 min. Antigen retrieval was done using heat induced epitopes retrieval (HIER) microwave oven method. Therefore the slides were cooked in 10 × concentrated citrate buffer (pH 6.0) (DCS Innovative Diagnostik-Systeme, Hamburg, Germany) for 30 min. After cooling down, the slides were covered with 3% hydrogen peroxide for 10 min. to block endogenous peroxidases activity followed by incubation with the specific primary antibodies anti-phospho-GSK3α/β (Y279/Y216) (#ab68476) (Abcam, Cambridge, UK) (1:100) and anti-phospho-GSK3α/β (Ser21/9) (#9331) (Cell Signaling, Danvers, MA, USA) (1:50) over night at 4° C. For negative control, antibody dilution buffer (DCS Innovative Diagnostik-Systeme) was used instead of the primary antibody. The conventional labeled-streptavidin-biotin method with horseradish-peroxidase and 3-amino ethylcarbazole as chromogen was used for detection according to the manufacturer’s instructions. Afterwards the slides were counterstained with Mayer’s Hematoxylin (Carl Roth, Karlsruhe, Germany) and mounted in Faramount Aquaeous Mounting Medium (Dako, Hamburg, Germany).

### MTT cytotoxicity assay

The cytotoxic effect of SB 216763 and DMSO was evaluated by MTT (3(4,5-Dimethyl-2-thiazolyl)-2,5-diphenyl-2H-tetrazolium bromide) assay. Therefore cells were seeded in 96-well plates in a density of 5000 cells/100 µl and cultured for 24 hours at 37° C under 5% CO_2_ and 95% air atmosphere. After incubation the medium was removed and cells were treated with SB 216763 (10 and 30 µM) and DMSO. Quadruplicates were used for each group. For negative control cells were only incubated with medium. After incubation the medium was replaced by 10 µl of 5 mg/ml MTT in 100 µl medium. MTT powder was purchased from Sigma-Aldrich (St. Louis, MO, USA). Cells were incubated then for additional 2 hours. Afterwards 100 µl MTT solubilisation solution (acidic isopropanol) was added to each well. The plates were shaken in the dark for 24 hours before absorbance was measured at 570 nm with background subtraction at 690 nm.

### Real time cell analyzer assay

To evaluate the cellular migration activity, the xCELLigence RTCA DP instrument (Roche Diagnostics, Mannheim, Germany) was used as described in the manufacturer´s instruction manual. Cells (60.000 cells/ well) were seeded in the upper chamber of CIM- Plate 16 (Roche Diagnostics). In order to establish an extracellular matrix, the underside of the upper chamber was coated with 30 µl of collagen type I (0.4 µg/µl) (Sigma-Aldrich, St. Louis, MO, USA) per well prior seeding. The cell indices were measured every 15 minutes for 18 hours. Each treatment condition was measured in triplicates.

### PCR array human epithelial to mesenchymal transition (EMT)

The Human Epithelial to Mesenchymal Transition (EMT) RT^2^ Profiler™ PCR Array was used according to the manufacturer’s instructions and profiles the expression of 84 key genes that either change their expression during this process or regulate those gene expression changes. The array includes cell surface receptor, extracellular matrix, and cytoskeletal genes mediating cell adhesion, migration, motility, and morphogenesis; genes controlling cell differentiation, development, growth, and proliferation; as well as signal transduction and transcription factor genes that cause EMT and all of its associated processes.

### *In vitro* scratch assay

To analyze cellular *in vitro* migration by using a scratch assay, cells were seeded in cell culture chamber slides and incubated at 37° C under 5% CO_2_ and 95% air atmosphere. When they reached 100% confluence, a straight artificial gap was created in the middle of the chamber by using a sterile 200 µl pipette tip. In order to smooth the edges of the scratch and remove floating cells, the cells were washed with medium several times. Afterwards the medium was removed and replaced by medium supplemented with 10 µM SB216763, 30 µM SB216763 or DMSO. Untreated cells were used as negative control. Photographs of the migrating cells were taken every 30 minutes by a microscope (Keyence, Osaka, Japan) with stage incubator (Tokai Hit, Shizuoka, Japan) as well as multipoint and time lapse function.

### Real-time PCR (RT-PCR) and statistical analysis

Indicated cell cultures were treated with the GSK3α/β inhibitor, SB 216763 (30µM) for 24 hours and then stimulated with the TLR agonists, LPS (2 µg/ml) and Poly (I:C) (10 µg/ml) for 2 hours. Total RNA was isolated using RNeasy plus Mini Kit (Qiagen) and real-time PCR (RT-PCR) was performed using a LightCycler1.5 (Roche). β-actin was used as an endogenous control and fold increase was calculated according to ΔΔCT method. TaqMan probes were used to detect the specific gene expression profiles of inflammatory cytokines Interleukin (IL)-1β, IL6, IL8, IL10, TNFα and IFNβ. Data are expressed as mean with standard deviation from three independent experiments. Statistical significance between the treated and untreated groups was evaluated by paired student’s *t*-test and the significant differences were represented as (^*^p < 0.05, ^**^p < 0.01, ^***^p < 0.001).

## References

[1] Luce D, Guenel P, Leclerc A, Brugere J, Point D, Rodriguez J (1988). Alcohol and tobacco consumption in cancer of the mouth, pharynx, and larynx: a study of 316 female patients. Laryngoscope.

[2] Leong SP, Cady B, Jablons DM, Garcia-Aguilar J, Reintgen D, Jakub J, Pendas S, Duhaime L, Cassell R, Gardner M, Giuliano R, Archie V, Calvin D (2006). Clinical patterns of metastasis. Cancer Metastasis Rev.

[3] Chin D, Boyle GM, Theile DR, Parsons PG, Coman WB (2004). Molecular introduction to head and neck cancer (HNSCC) carcinogenesis. Br J Plast Surg.

[4] Douglas WG, Tracy E, Tan D, Yu J, Hicks WL, Rigual NR, Loree TR, Wang Y, Baumann H (2004). Development of head and neck squamous cell carcinoma is associated with altered cytokine responsiveness. Mol Cancer Res.

[5] Pries R, Wollenberg B (2006). Cytokines in head and neck cancer. Cytokine Growth Factor Rev.

[6] Pries R, Nitsch S, Wollenberg B (2006). Role of cytokines in head and neck squamous cell carcinoma. Expert Rev Anticancer Ther.

[7] Patel S, Woodgett J (2008). Glycogen synthase kinase-3 and cancer: good cop, bad cop?. Cancer Cell.

[8] Martin M, Rehani K, Jope RS, Michalek SM (2005). Toll-like receptor-mediated cytokine production is differentially regulated by glycogen synthase kinase 3. Nat Immunol.

[9] Hofmann C, Dunger N, Schölmerich J, Falk W, Obermeier F (2010). Glycogen synthase kinase 3-β: a master regulator of toll-like receptor-mediated chronic intestinal inflammation. Inflamm Bowel Dis.

[10] Woodgett JR (1990). Molecular cloning and expression of glycogen synthase kinase-3/factor A. EMBO J.

[11] Woodgett JR, Plyte SE, Pulverer BJ, Mitchell JA, Hughes K (1993). Roles of glycogen synthase kinase-3 in signal transduction. Biochem Soc Trans.

[12] Woodgett JR (2001). Judging a protein by more than its name: GSK-3. Sci STKE.

[13] Kaidanovich-Beilin O, Woodgett JR (2011). GSK-3: Functional Insights from Cell Biology and Animal Models. Front Mol Neurosci.

[14] Embi N, Rylatt DB, Cohen P (1980). Glycogen synthase kinase-3 from rabbit skeletal muscle. Separation from cyclic-AMP-dependent protein kinase and phosphorylase kinase. Eur J Biochem.

[15] Harwood AJ, Plyte SE, Woodgett J, Strutt H, Kay RR (1995). Glycogen synthase kinase 3 regulates cell fate in Dictyostelium. Cell.

[16] He X, Saint-Jeannet JP, Woodgett JR, Varmus HE, Dawid IB (1995). Glycogen synthase kinase-3 and dorsoventral patterning in Xenopus embryos. Nature.

[17] Kaidanovich-Beilin O, Lipina TV, Takao K, van Eede M, Hattori S, Laliberté C, Khan M, Okamoto K, Chambers JW, Fletcher PJ, MacAulay K, Doble BW, Henkelman M (2009). Abnormalities in brain structure and behavior in GSK-3alpha mutant mice. Mol Brain.

[18] Kockeritz L, Doble B, Patel S, Woodgett JR (2006). Glycogen synthase kinase-3—an overview of an over-achieving protein kinase. Curr Drug Targets.

[19] Jope RS, Johnson GV (2004). The glamour and gloom of glycogen synthase kinase-3. Trends Biochem Sci.

[20] Jope RS, Yuskaitis CJ, Beurel E (2007). Glycogen synthase kinase-3 (GSK3): inflammation, diseases, and therapeutics. Neurochem Res.

[21] Doble BW, Patel S, Wood GA, Kockeritz LK, Woodgett JR (2007). Functional redundancy of GSK-3alpha and GSK-3beta in Wnt/beta-catenin signaling shown by using an allelic series of embryonic stem cell lines. Dev Cell.

[22] Doble BW, Woodgett JR (2007). Role of glycogen synthase kinase-3 in cell fate and epithelial-mesenchymal transitions. Cells Tissues Organs.

[23] Doble BW, Woodgett JR (2003). GSK-3: tricks of the trade for a multi-tasking kinase. J Cell Sci.

[24] Hughes K, Nikolakaki E, Plyte SE, Totty NF, Woodgett JR (1993). Modulation of the glycogen synthase kinase-3 family by tyrosine phosphorylation. EMBO J.

[25] Beurel E, Jope RS (2006). The paradoxical pro- and anti-apoptotic actions of GSK3 in the intrinsic and extrinsic apoptosis signaling pathways. Prog Neurobiol.

[26] Matsuda T, Zhai P, Maejima Y, Hong C, Gao S, Tian B, Goto K, Takagi H, Tamamori-Adachi M, Kitajima S, Sadoshima J (2008). Distinct roles of GSK-3alpha and GSK-3beta phosphorylation in the heart under pressure overload. Proc Natl Acad Sci USA.

[27] Cao Q, Lu X, Feng YJ (2006). Glycogen synthase kinase-3beta positively regulates the proliferation of human ovarian cancer cells. Cell Res.

[28] Ghosh JC, Altieri DC (2005). Activation of p53-dependent apoptosis by acute ablation of glycogen synthase kinase-3beta in colorectal cancer cells. Clin Cancer Res.

[29] Zhou Y, Uddin S, Zimmerman T, Kang JA, Ulaszek J, Wickrema A (2008). Growth control of multiple myeloma cells through inhibition of glycogen synthase kinase-3. Leuk Lymphoma.

[30] Wang L, Lin HK, Hu YC, Xie S, Yang L, Chang C (2004). Suppression of androgen receptor-mediated transactivation and cell growth by the glycogen synthase kinase 3 beta in prostate cells. J Biol Chem.

[31] Ougolkov AV, Fernandez-Zapico ME, Savoy DN, Urrutia RA, Billadeau DD (2005). Glycogen synthase kinase-3beta participates in nuclear factor kappaB-mediated gene transcription and cell survival in pancreatic cancer cells. Cancer Res.

[32] Dickey A, Schleicher S, Leahy K, Hu R, Hallahan D, Thotala DK (2011). GSK-3β inhibition promotes cell death, apoptosis, and *in vivo* tumor growth delay in neuroblastoma Neuro-2A cell line. J Neurooncol.

[33] Korur S, Huber RM, Sivasankaran B, Petrich M, Morin P, Hemmings BA, Merlo A, Lino MM (2009). GSK3beta regulates differentiation and growth arrest in glioblastoma. PLoS One.

[34] Wang H, Kumar A, Lamont RJ, Scott DA (2014). GSK3β and the control of infectious bacterial diseases. Trends Microbiol.

[35] Hill EV, Ng TH, Burton BR, Oakley CM, Malik K, Wraith DC (2015). Glycogen synthase kinase-3 controls IL-10 expression in CD4(+) effector T-cell subsets through epigenetic modification of the IL-10 promoter. Eur J Immunol.

[36] Khan KA, Dô F, Marineau A, Doyon P, Clément JF, Woodgett JR, Doble BW, Servant MJ (2015). Fine-Tuning of the RIG-I-Like Receptor/Interferon Regulatory Factor 3-Dependent Antiviral Innate Immune Response by the Glycogen Synthase Kinase 3/β-Catenin Pathway. Mol Cell Biol.

[37] Wang H, Garcia CA, Rehani K, Cekic C, Alard P, Kinane DF, Mitchell T, Martin M (2008). IFN-beta production by TLR4-stimulated innate immune cells is negatively regulated by GSK3-beta. J Immunol.

[38] Gammon L, Mackenzie IC (2016). Roles of hypoxia, stem cells and epithelial-mesenchymal transition in the spread and treatment resistance of head and neck cancer. J Oral Pathol Med.

